# First Case of Tularemia Reported in Portugal: Probably of Imported Origin

**DOI:** 10.3389/fpubh.2018.00325

**Published:** 2018-11-19

**Authors:** Isabel Lopes de Carvalho, Paula Nascimento, Maria Sofia Núncio, Miguel Toscano Rico

**Affiliations:** ^1^Infectious Department, National Institute of Health Dr. Ricardo Jorge, Lisbon, Portugal; ^2^Internal Medicine Service 4, Centro Hospitalar de Lisboa Central, Santa Marta Hospital, Lisbon, Portugal

**Keywords:** tularemia, patient, ulceroglandular, *Francisella tularensis* subsp. *holarctica*, case report, Portugal

## Abstract

The authors report the case of a 47-year-old man who walked in the countryside on the island of Bornholm, during the summer period. Three days later, fever, myalgias and adynamia began. The serological tests, Real-time PCR and isolation of the bacteria from the culture of lymph biopsy confirmed the presence of *Francisella tularensis* subsp. *holarctica*.

## Introduction

Tularemia is a zoonotic disease caused by *Francisella tularensis*, a gram-negative, facultative intracellular bacterium. Typically, human and animal infections are caused by *F. tularensis* subspecies *tularensis* (type A) strains mainly in Canada and USA, and *F. tularensis* subspecies *holarctica* (type B) strains throughout the northern hemisphere, including Europe (1, 2).

Tularemia is a disease with epidemiological surveillance in Europe since 2003 (Decision 2000/96/EC) (3). Despite being considered an uncommon disease, recent outbreaks have been reported in several countries, including Spain, France, Scandinavia, Balkans and Hungary, and sporadic cases in Austria, Italy and the United Kingdom (4).

In spite offbeing a disease of compulsory declaration in Portugal since 2003, until now no human cases were reported.

In Portugal, seroprevalence rate of high-risk population is 8.9% (5) and *F. tularensis* subsp. *holarctica* was first detected in 2007 by molecular methods in a human sample (6). Since 1998, National Institute of Health has provided the laboratory diagnosis of this disease, following the occurrence of an epidemic outbreak in Spain.

The authors report here the first case of ulceroglandular form of tularemia in Portugal, probably of imported origin.

## Case report

This study was carried out in accordance with the recommendations of the guidelines of Helsinki Committee. A written informed consent was obtained from the patient for publication of this case report.

A previously healthy 47-years-old male developed abruptly malaise, high fever (40°C) and chills only 3 days after walking and sleeping in the countryside of the island of Bornholm (Denmark) during summer season. He also reported profuse night sweats, a small left infraclavicular non-painful cutaneous lesion. The patient did not report outdoor activities in the month before and did not recall any tick bite. Three days later the fever vanished and he was first observed in a hospital in Berlin. He was prescribed with amoxicillin/clavulanate 875mg/125mg twice-a-day for 7 days.

On the 15th day of disease, he was observed in Portugal because of ongoing malaise. During the observation he did not complain of respiratory symptoms nor headache.

**Figure 1:**
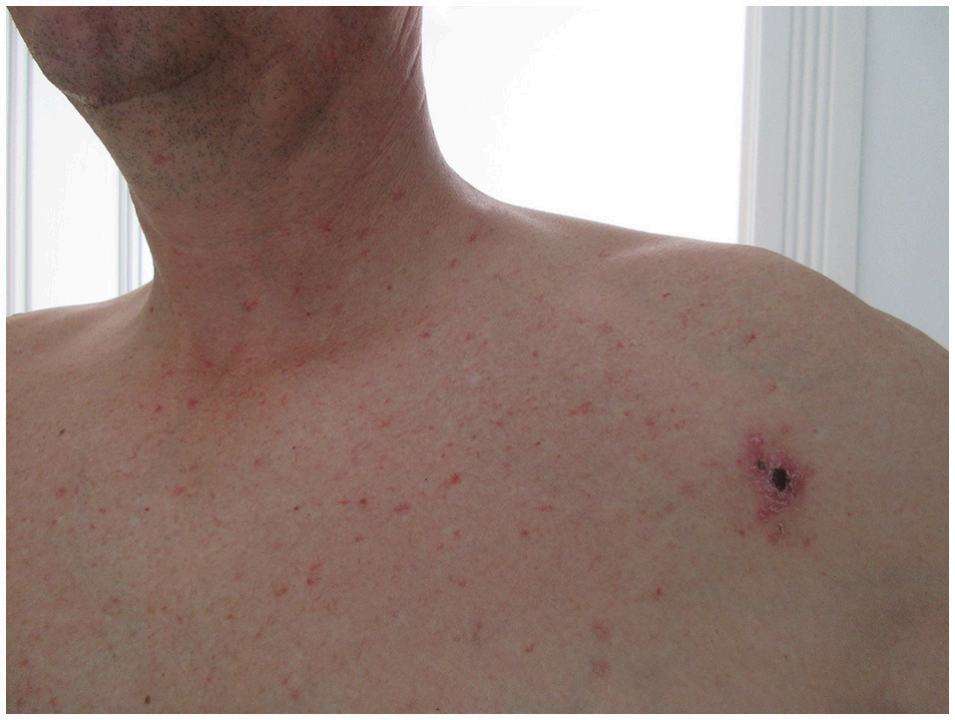
Eschar of inoculation and Rash taken on the 15th day.

**Figure 2:**
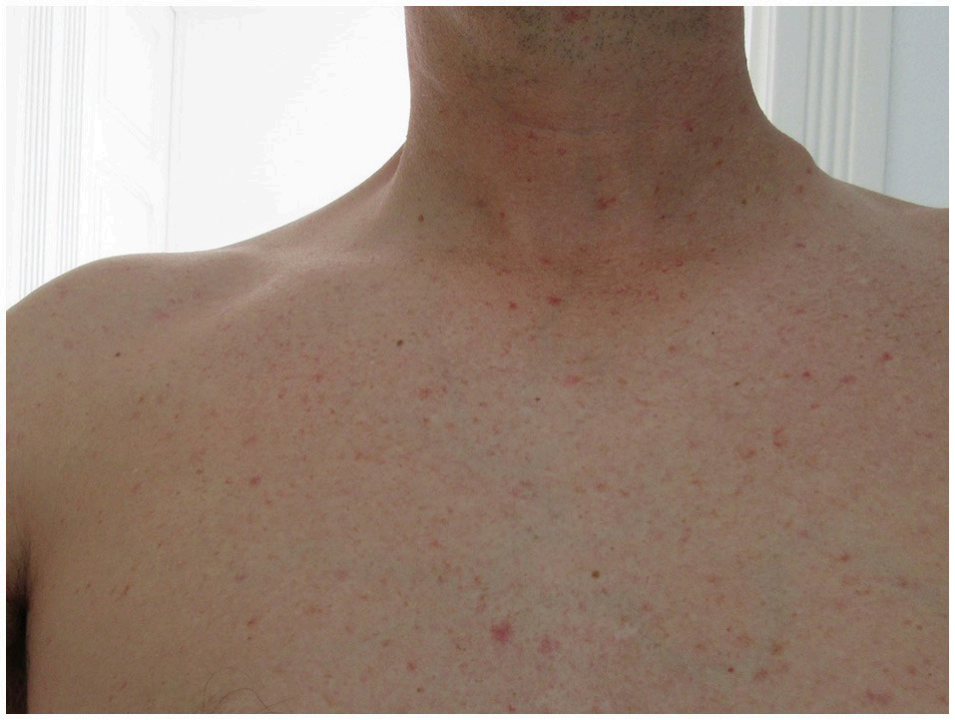
Left cervical adenopathy taken on the 15th day.

He presented a non-pruritic macular erythematous rash of the trunk, a non-painful left infraclavicular cutaneous lesion covered with a black crust suggestive of a skin eschar and multiple small painless, non-adherent cervical, and axillar lymphadenopathies (Figures 1, 2).

Routine laboratory tests evidenced minor unspecific inflammatory abnormalities: white blood count (WBC; leucocytes 12.060 × 10^6^/L, polymorphonuclear predominancy), platelet count 307000 × 10^6^/L, C-reactive protein 28.4 mg/L. Liver functions tests, blood urea nitrogen, creatinine and electrolyte levels, were within normal range. Human immunodeficiency virus infection tested negative.

Soft tissue ultrasound revealed several lymphadenopathies of the left axillary region, most within dimensions of 1.0–1.5cm (of the largest axis) and two larger (1.8 and 2.3cm) with tendency to suppuration.

Considering an exanthematic fever and an inoculation-like lesion, a *Rickettsial* infection or Lyme borreliosis were suspected. Blood samples were collected and the patient initiated empirically doxycycline 100 mg bid (twice a day). Later on both detections for antibodies to *Rickettsia* sp. and *Borrelia burgdorferi* s.l were negative. Careful revision of zoonosis with prominent lymphadenopathies in Denmark was carried out and an infection with *Francisella tularensis* was suspected.

Serum specimen was shown to be positive for *F. tularensis* specific antibodies, which was demonstrated by an agglutination test with a titer of 160 (7), followed by enzyme-linked immunosorbent assay (ELISA) classic *Francisella tularensis* (Virion/Serion GmbH Institute, Würzburg, Germany) IgM (titer: 114.53 U/ml (cut-off 15 U/ml), and IgG (titer: > 300 U/ml (cut off 15 U/ml).

As the patient showed no improvement after 4 days with doxicycline he started intramuscular streptomycin 1 g every 12 h in an outpatient basis for 10 days without complications.

The inoculation-like lesion resolved, but the lymphadenopathies remained mostly unchanged. Despite this, the patient left Portugal for working reasons.

Three weeks later the left cervical lymphadenopathy increased in size and the axillary evolved into a suppurative lymphadenitis. He was called back and an excision of the left cervical lymph node was performed.

The excisional biopsy of the lymph node was cultured on chocolate agar media (bioMérieux) at 37°C in a 5% CO_2_- enriched atmosphere of (8). After 3 days, some suspicious colonies of *Francisella* grow. This observation was confirmed by a real-time multitarget TaqMan PCR, using *tul4* and *ISFtu2* assays (9) and *fopA* gene (10) with positives further tested using real-time TaqMan PCR assays which differentiate between *F. tularensis* subsp. *tularensis* (type A) and *F. tularensis* subsp. *holarctica* (type B) (11). For additional characterization, a phylogenetically informative region of *lpnA* (231 bp) was amplified by conventional PCR as previous described (12).

Amplicons obtained by *lpnA* PCR were purified and sequenced with the ABI BigDye Terminator Cycle Sequencing Ready Reaction Kit (Applied Biosystems, Inc., Foster City, CA, USA). The *lpnA* sequences for *F. tularensis* subsp. *holarctica* (PoHuF2) was assigned with the GenBank accession number: MH068785.

On the other hand, histopathology showed cell necrosis and no evidence of tumor cells nor *Mycobacterium tuberculosis* bacilli was found. An ulceroglandular form of tularemia was unequivocally diagnosed.

In the absence of response to the first line antibiotic therapy with aminoglycosides, he was treated with ciprofloxacin 500 mg twice-a-day for 60 days and performed percutaneous drainage of the bigger axillar lymph nodes with clinical improvement.

## Discussion

Clinical presentation of tularemia is poorly specific and clinical signs vary according with its six main clinical presentation forms: ulceroglandular, glandular, oculoglandular, oropharyngeal, pneumonic and typhoidal (1, 13, 14).

Our patient presented with a typical ulceroglandular form and as frequently described, he developed a suppurative lymphadenitis, requiring surgical drainage and lymph node resection.

The European Center for Disease Control and Prevention (ECDC) 2016 surveillance report refers 526 confirmed cases in a number of European countries in 2014, with Sweden reporting the highest case rate (3). Although there are no reports of tularemia for Denmark in 2003 a confirmed case of human was recorded (15). Furthermore in 2001, two cases of tularemia were reported after an orienteering contest on the Island of Bornholm, at the same location where we consider the transmission of infection occurred for the present case (16). The case here presented highlights the role of ticks as vectors in transmission of tularemia, since the patient showed an eschar of inoculation. Moreover, commonly the infection occurs during warm seasons in adult men. Ulcer and lymphadenopathies are reported in the upper and lower parts of the body when infections are caused by arthropods, as happen in the present case (1).

Tularemia is often a prolonged and debilitating disease. Three cases of tick-borne transmission between 2012 and 2013 have been reported in Germany (17), data from France point out that more than 10% of reported human infections of *F. tularensis* is derived by tick transmission (13). In Switzerland, three probable cases of tick-borne tularemia were described in 2013 (17). In Portugal until now no human cases were reported despite the detection of *F. tularensis* subsp. *holarctica* in different tick species, lagomorphs and in one human sample (6, 18). In humans tularemia probably represents a re-emerging disease with a high proportion of undiagnosed cases (19). Our report represents the first imported tularemia case notified in Portugal.

Despite aminoglycosides remains the treatment of choice for severe forms, fluoroquinolones and doxycycline may be appropriate for the less severe ones. Failures in empirical therapy are described, but mostly occur because of the delay in treatment with the appropriate antibiotic. Some reports suggest that fluoroquinolones are associated with lower relapse rates, particularly in patients with extensive lymph node involvement, for which a 2–3 week treatment course is usually insufficient for cure (1, 14).

Delay in initiating appropriate treatment (around 2 weeks) and extensive lymphadenopathy involvement might have contributed to streptomycin failure and should have favored a quinolone-basis treatment instead. We decided for a 2-months ciprofloxacin treatment due to the persistence of documented viable bacteria in cervical ganglion and markers of cure are not available so far.

To our best knowledge, this was the first notified case of tularemia in Portugal of highly probable imported origin. The increasing mobility of travelers and the potential for exposure to diseases outside their home countries poses clinicians an increased effort to know the epidemiological background of the patients.

The present case illustrates the importance to include tularemia in the differential diagnosis of patients with fever combined with a lymphadenopathy (ulcerative or not) and to initiate early laboratory diagnosis of suspicious cases. Moreover, good communication between clinicians and the reference laboratory was important to achieve the diagnosis of this patient.

## Author contributions

ILC and MSN serotyped and identified the strain. MTR and PN evaluated and analyzed the clinical symptoms and history of the patient. ILC and MTR wrote the manuscript.

### Conflict of interest statement

The authors declare that the research was conducted in the absence of any commercial or financial relationships that could be construed as a potential conflict of interest.
